# A clinical microscopy dataset to develop a deep learning diagnostic test for urinary tract infection

**DOI:** 10.1038/s41597-024-02975-0

**Published:** 2024-02-01

**Authors:** Natasha Liou, Trina De, Adrian Urbanski, Catherine Chieng, Qingyang Kong, Anna L. David, Rajvinder Khasriya, Artur Yakimovich, Harry Horsley

**Affiliations:** 1 Bladder Infection and Immunity Group (BIIG), UCL Centre for Kidney & Bladder Health, Division of Medicine, University College London, Royal Free Hospital Campus, London, UK; 2 UCL EGA Institute for Women’s Health, Faculty of Population Health Sciences, University College London, London, UK; 3 Center for Advanced Systems Understanding (CASUS), Görlitz, Germany; 4 Helmholtz-Zentrum Dresden-Rossendorf e. V. (HZDR), Dresden, Germany; 5 Department of Microbial Diseases, Eastman Dental Institute (EDI), University College London, London, UK; 6 Institute of Computer Science, University of Wrocław, Wrocław, Poland

**Keywords:** Diagnostic markers, Urinary tract infection, Medical imaging, Translational research

## Abstract

Urinary tract infection (UTI) is a common disorder. Its diagnosis can be made by microscopic examination of voided urine for markers of infection. This manual technique is technically difficult, time-consuming and prone to inter-observer errors. The application of computer vision to this domain has been slow due to the lack of a clinical image dataset from UTI patients. We present an open dataset containing 300 images and 3,562 manually annotated urinary cells labelled into seven classes of clinically significant cell types. It is an enriched dataset acquired from the unstained and untreated urine of patients with symptomatic UTI using a simple imaging system. We demonstrate that this dataset can be used to train a Patch U-Net, a novel deep learning architecture with a random patch generator to recognise urinary cells. Our hope is, with this dataset, UTI diagnosis will be made possible in nearly all clinical settings by using a simple imaging system which leverages advanced machine learning techniques.

## Background & Summary

UTI can often be clinically identified by the presence of lower urinary tract symptoms (LUTS), with the classical symptoms being burning or pain on urination and frequency of urination. UTIs are the most common bacterial infection in humans with the potential to become a recurrent infection or lead to life-threatening infections and sepsis^1^. Women are not only at increased risk of UTI, but also more likely to develop complicated infections^2^. Not surprisingly, UTIs are associated with a substantial health and economic burden^3^ and the prevalence of antibiotic prescriptions and hospital admissions related to urine infections is on the rise^4,5^.

Rapid identification of infection and timely administration of antimicrobial treatment can prevent adverse complications. Point-of-care testing (POCT), tests which are performed at the bedside at the time and place of patient care, is the preferred diagnostic practice^6^. However, the current routine tests, namely the urine dipstick and midstream urine culture, are inadequate to detect UTI^7,8^. Without an accurate POCT, clinicians are ill equipped to diagnose infections, thus contributing to inappropriate antibiotic use and potentially driving antimicrobial resistance^9^.

Urine microscopy and identification of urinary cells from freshly voided urine is an alternative POCT with greater sensitivity than both aforementioned methods. The presence of white blood cells (WBC, or pyuria) in an unspun, unstained specimen of urine examined shortly after void is particularly predictive of a UTI^10,11^. The presence of epithelial cells (EPC) is also suggestive of infection as urinary epithelial cells are actively involved in antibacterial activities^12–14^.

**Fig. 1:**
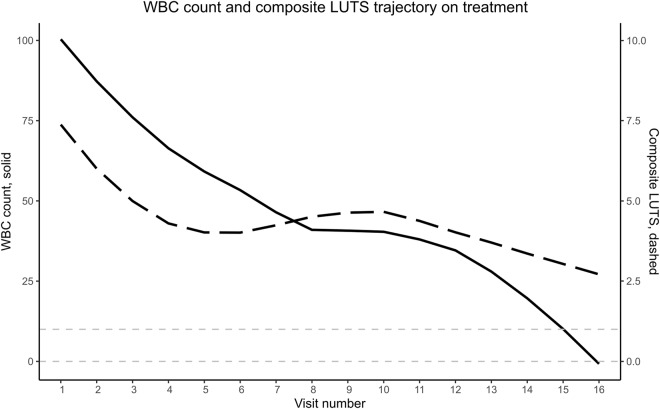
WBC count and composite LUTS trajectory on treatment. WBC count (solid line) as measured by urine microscopy correlates with a composite score of LUTS (dashed line) over the course of antimicrobial treatment. By the final clinical attendances, WBC count drops within a physiological WBC range as marked by the pale dashed grey lines.

Urinary microscopy measures and explores the host immune response and, therefore, accurately reflects the underlying pathophysiological state of the urinary tract. Use of this test has been shown to improve patient outcomes^15^. Pain, storage, and voiding symptoms have been found to be the most reliable predictors of microscopic pyuria, and in turn correlate with measures of quality of life. In our experience, treating patients with chronic UTI, we find that peak symptoms coincide with peak cell counts (Fig. 1). Unfortunately, while the benefits of urine microscopy have clear clinical benefits, it requires the time and manual labour of an experienced microscopist thus limiting its availability as a POCT to highly specialised clinics in well-developed countries^11^.

Machine learning in biomedical imaging is increasingly used as an adjunct to enhance or automate conventional diagnostics. An image dataset of spun urinary sediment to identify three urinary cell types has been produced to automate urinalysis and detect a wide range of urinary and kidney diseases from hospitalised patients^16^. Furthermore, nano-resolution microscopy images of urine cells have been applied to detect bladder cancer^17^. However, a clinically relevant and representative dataset of urinary cells obtained from symptomatic patients with urine infection does not currently exist. This is due to the wide range and often equivocal nature of cellular content in symptomatic patients. While these difficulties might be overcome by processing the urine to produce urinary sediment^16^, or the application of advanced imaging techniques^17^ or histological stains^18^, such a model could no longer be offered as a POCT as the workflow would require access to specialised equipment found in large, centralised laboratories away from the point of patient care. Moreover, such advanced equipment and techniques are likely only to be available in well developed countries. There is no open dataset of high-quality urinary cells annotated for the analytical task of UTI detection to date.

We have produced an open image dataset of urinary cells which can be used to identify markers of infection using machine learning techniques. Our image dataset of voided urine is clinically representative of patients with known urine infection. Unlike other cellular image datasets, cell identification techniques such as histological staining have not been deployed and therefore no laboratory processing is required. This was purposefully done with the ambition of creating an accurate POCT using a simple imaging system which leverages machine learning.

## Methods

### Ethics

Written informed consent was obtained from all participants in accordance with Good Clinical Practice guidance and participants agreed to the open publication of data. Ethics was approved by Health Research Authority (HRA) and Health and Care Research Wales (HCRW) under “A prospective observational cohort study of the pathophysiology of urinary tract infection”, IRAS 295252, protocol number 143470, and REC reference 22/WA/0069.

### Clinical samples

300 urine samples were randomly obtained from patients with symptomatic UTI from the Whittington Health NHS Trust in London, UK. LUTS data was collected using a validated 39-question in inventory grouped into pain, urgency, voiding, and stress symptoms and assessed in binary yes or no response (Supplementary File 1). Frequency of urination and incontinence during the day and night was also assessed.

### Data acquisition

Urine samples were collected as natural voids and processed on-site within one hour to limit cellular degradation. Brightfield microscopic examination (Olympus BX41F microscope frame, U-5RE quintuple nosepiece, U-LS30 LED illuminator, U-AC Abbe condenser) was performed using a x20 objective (Olympus PLCN20x Plan C N Achromat 20x/0.4). A disposable haemocytometer (C Chip™) was used for enumeration of red blood cells (RBC), white blood cells (WBC), epithelial cells (EPC), and the presence of all other relevant cellular content per 1 µl of urine by two experienced microscopists.

Images were acquired using the aforementioned brightfield microscope using a 0.5X C-mount adapter coupled to a digital scientific colour camera (Infinity 3S-1UR, Teledyne Lumenera). Images were taken in 16-bit colour in 1392 × 1040 TIFF format using Micromanager software^19^. Daily Kohler illumination and global white balance was performed to ensure consistency in image acquisition. An enriched dataset approach was taken to maximise urinary cellular content in the acquired images. Such data curation was also necessary to attenuate object sparsity.

### Dataset annotation

300 images were acquired and manually annotated to produce 3,562 objects by first identifying cells of interest as a binary semantic segmentation task. Individual pixels were dichotomously labelled as either informative objects, foreground, or non-informative background. Non-informative background was further constrained by including unidentifiable cells, such as debris or grossly out of focus particles. Binary annotation was initially performed using ilastik^20^, an open source software using a Random Forest classifier for pixel classification, then manually refined at the pixel level to ensure accurate segmentation. This produced a binary mask in 1392 × 1040 TIFF format with values [0,1] for each corresponding raw colour image.

All 3,562 objects, or cells of interest, were subsequently labelled manually by two expert microscopists into one of seven clinically significant multi-class categories: rods, RBC/WBC, yeast, miscellaneous, single EPC, small EPC sheet, and large EPC sheet (Table 1). This produced a multi-class mask in 1392 × 1040 TIFF format with integral values between [0,7].

**Table 1:** Data structure.

Folder	Files	Objects	Count	Pixel Values
img	300	Raw data		0-65535
bin_mask	300	Background/Foreground		0/1
mult_mask	300	Background/Class		0
		Rod	1697	1
		RBC/WBC	1056	2
		Yeast	41	3
		Miscellaneous	550	4
		Single EPC	182	5
		Small EPC sheet	26	6
		Large EPC sheet	10	7
		Total	3562	

These classes were chosen due to their clinical significance. Coliform bacteria are frequently implicated in UTI pathogenesis and are rod-shaped with each cell unit measuring 0.25–1.0 μm in width and 2.0 μm in length. These bacteria can elongate up to 15μm to produce a filamentous morphology, a phenomenon often associated with bacterial pathogenicity in the urinary tract^21,22^. Yeast (most commonly of the *Candida* species) are also seen in urine, and may represent a commensal organism or infectious pathogen^23^. Their size is dependent on their mitotic state, and in certain states may be confused with erythrocytes. RBC and WBC, haematuria and pyuria respectively, are cellular indicators of infection^24,25^. EPC are often seen as individual cells or sheets of cells. A powerful mechanism to rapidly reduce bacterial load is to shed the superficial bladder epithelium invaded and colonised by bacteria^12^. The presence of large EPC sheets may therefore indicate more widespread infection hence more extreme cellular exfoliation. Work is ongoing to further subtype the aforementioned classes (e.g. distinct WBC populations such as macrophages and lymphocytes) and annotate new classes (e.g. cocci, another bacterial morphology).

### Data preprocessing

First, the image was rescaled according to the scale factor either 0.2, 0.3, 0.5 or 1, and thereby, if applicable, decreasing its resolution. This allowed the model to analyse a larger area while keeping the patch size uniform, an important strategy in the case of sparse data. Then, 256 × 256 patches were cut from a random region in the image. Finally, all values within the patch were rescaled to fall within the range of [−1, 1] by performing the following operations: divide by 255, the highest potential value, then multiply by 2, and finally subtract 1. In the case of training data, random vertical and horizontal flips were performed to increase the variation in the data and encourage model generalisation.

### Patch U-Net architecture

Generally, we followed the architectures described here^26,27^. There were, however, a few notable changes. Firstly, we added instance normalisation layers^28^. Secondly, we made the size of a network scalable by specifying the number of channels produced by the initial convolutional layer.

Similarly to the architecture proposed by Ronneberger and colleagues^26^, our network consisted of an encoder (contracting) and decoder (expansive) path. A critical component of the network was the convolutional block^29^, which consisted of repeated applications of 3 × 3 convolutions, each followed by batch normalisation^30^ and rectified linear unit (ReLU)^29^. The contracting path consisted of 5 convolutional blocks, each followed by an instance normalisation layer and a 2 × 2 max pooling operation^29^ with stride 2 for downsampling. After each downsampling step, we doubled the number of feature channels.

Every step in the expansive path consisted of an upsampling of the feature map followed by a 2 × 2 convolution which halved the number of feature channels, followed by a convolutional block and instance normalisation layer.

### Loss functions

The loss function was computed by a pixel-wise sigmoid over the final feature map with the combined binary cross entropy^31^ and Dice coefficient loss function^32,33^.

The sigmoid function^34^ is defined as:

1 $$\sigma \left({\boldsymbol{x}}\right)=\frac{1}{1+{{\boldsymbol{e}}}^{-{\boldsymbol{x}}}},$$

and casts the prediction values into (0,1) range. Let’s define *Y* as ground truth, $\widehat{Y}$ as model prediction, and *N* as the number of pixels. The cross-entropy penalises^31^ the deviation from the ground truth at each position using:

2 $$BCE(Y,\widehat{Y})=-\frac{1}{N}{\sum }_{i=1}^{N}({y}_{i}.log({\widehat{y}}_{i})+(1-{y}_{i}).log(1-{\widehat{y}}_{i}))$$

This is combined with Dice loss^32,33^, which is defined as:

3 $$DL\,(Y,\widehat{Y})\,=1-D\,(Y,\widehat{Y})\,=1-\frac{2.| Y\cap \widehat{Y}| \,}{\,| Y| \,+\,| \widehat{Y}| \,},$$

where $D\left(Y,\widehat{Y}\right)$ is defined in Eq. (1). The final loss function is defined as:

4 $$L(Y,\widehat{Y})=\alpha .BCE(Y,\widehat{Y})+\beta .DL(Y,\widehat{Y})$$

It is calculated across the batch to make it more stable. In our experiments, we set ***α*** = ***β*** = **1**.

### Batch generation

To prepare batches of training data for the Patch U-Net, full-scale images were dynamically pre-processed into patches of 256 × 256 pixels during the training. Training of the Patch U-Net was performed on mini-batches of such patches. Given the sparsity of the objects in the images, a procedure evaluating emptiness of the image was devised. As a result, for each mini-batch, patches were generated using the following procedure:

1. Choose a random value as
  $$h \sim U\left\{0,H-1\right\}$$
  where *H* is the height of the images
2. Choose a random value as
  $$w \sim U\left\{0,W-1\right\}$$
  where *W* is the width of the images and *U* represents a Discrete Uniform Distribution^35^
3. Get patches from the image *X* as follows:
  $$X{\prime\prime} \equiv X\left[h,h+\Delta h:w,w+\Delta w\right],\Delta h=\Delta w$$
4. Get batches of patches as above as follows:

$$B=\{{X{\prime\prime} }_{1},{X{\prime\prime} }_{2},\ldots ,{X{\prime\prime} }_{BS}\}s.t.\frac{1\times 100}{BS\times H\times W}{\sum }_{i=1}^{BS}{\sum }_{x}{\sum }_{y}{X{\prime\prime} }_{i,bw}(x,y)$$

where *BS* is the batch size and ${X{\prime\prime} }_{i,bw}$ is the binary mask corresponding to the *i*^*th*^ image patch in the batch *B*.

### Metrics

To evaluate model performance during training we employed the Sørensen–Dice coefficient^32,33^ which measures the ratio between the area of overlap and the total number of pixels classified as foreground in both images and is described by Eq. (1):

5 $$D(Y,\widehat{Y})=\frac{2.| Y\cap \widehat{Y}| }{| Y| +| \widehat{Y}| },$$

where $\widehat{Y}$ is the segmentation mask returned by the model, and *Y* is the ground truth.

### Training evaluation

During training, the performance on both train and validation sets was calculated mini-batch wise where *b,h,w* respectively correspond to the index of the sample in a mini-batch and the position of a pixel in the sample respectively. *Y* and $\widehat{Y}$ are as defined previously.

6 $${D}_{train}(Y,\widehat{Y})=\frac{2.{\sum }_{b,h,w}{Y}_{b,h,w}.{\widehat{Y}}_{b,h.w}}{{\sum }_{b,h,w}{Y}_{b,h,w}+{\sum }_{b,h,w}{\widehat{Y}}_{b,h.w}},$$

Such an approach allowed us to address the sparse patches, i.e. patches where only a few pixels were marked as foreground. Such patches could contribute unrealistically high performance, should the metric be calculated in a sample-wise manner. In contrast, our approach allowed us to alleviate such circumstances, ensuring better training performance.

### Testing evaluation

For the final evaluation, we opted to emulate the real-world inference, and thus the metrics were computed image-wise. Since our model was patch-based, each image was split into patches prior to inputting into the model. To avoid potential issues at the edges of each patch, inference was performed on overlapping patches. Next, predictions were combined into a final mask by means of taking maximum from overlapping regions. The following metrics described in Eqs. (7)–(13) were used for evaluation.

7 $${D}_{test}(Y,\widehat{Y})=\frac{1}{n}{\sum }_{i\in \{1,...,n\}}\frac{2.{\sum }_{h,w}{Y}_{i,h,w}.{\widehat{Y}}_{i,h.w}}{{\sum }_{h,w}{Y}_{i,h,w}+{\sum }_{h,w}{\widehat{Y}}_{i,h,w}},$$

where $h,w,Y,\widehat{Y}$ have the same definition as in Eq. (6) and *i* is the image index in the test set. This was reported as the Dice coefficient^32,33^ in Table 2.

8 $$Io{U}_{test}({\boldsymbol{Y}},\widehat{{\boldsymbol{Y}}})=\frac{1}{n}{\sum }_{i\in \{1,...,n\}}\frac{{\sum }_{h,w}{Y}_{i,h,w}.{\widehat{Y{\prime} }}_{i,h.w}}{{\sum }_{h,w}{Y}_{i,h,w}+{\sum }_{h,w}{\widehat{Y{\prime} }}_{i,h,w}},$$

where *h,w,Y,i* have the same definition as in Eq. (7) and $\widehat{Y{\prime} }$ is the model prediction after binarizing to {0,1} based on a threshold, here 0.5. This was reported as *IoU*^36^ in Table 2.

9 $${P}_{test}(Y,\widehat{Y})=\frac{1}{n}{\sum }_{i\in \{1,...,n\}}\frac{{\sum }_{h,w}{Y}_{i,h,w}.{\widehat{Y{\prime} }}_{i,h.w}}{{\sum }_{h,w}{Y}_{i,h,w}.{\widehat{Y{\prime} }}_{i,h.w}+{\sum }_{h,w}{Y}_{i,h,w}^{\perp }\;.{\widehat{Y{\prime} }}_{i,h,w}}$$

where $h,w,Y,i,\widehat{{\boldsymbol{Y}}{\prime} }$ have the same definition as in Eq. (8) and ${{\boldsymbol{Y}}}^{\perp }$ is defined as the ground truth, wherein the binary encoding convention has been inverted, such that the logical values of 0 and 1 are interchanged. This was reported as *Precision*^37^ in Table 2.

10 $${R}_{test}(Y,\widehat{Y})=\frac{1}{n}{\sum }_{i\in \{1,...,n\}}\frac{{\sum }_{h,w}{Y}_{i,h,w}\;.{\widehat{Y{\prime} }}_{i,h.w}}{{\sum }_{h,w}{Y}_{i,h,w}\;.{\widehat{Y{\prime} }}_{i,h.w}+{\sum }_{h,w}{Y}_{i,h,w}\;.{\widehat{Y}{\prime} }_{i,h,w}^{\perp }}$$

where $h,w,Y,i,\widehat{{\boldsymbol{Y}}{\prime} }$ have the same definition as in Eq. (9) and ${\widehat{{\boldsymbol{Y}}}{\prime} }^{\perp }$ is defined as the model prediction, wherein the binary encoding convention has been inverted, such that the logical values of 0 and 1 are interchanged. This was reported as *Recall*^37^ in Table 2.

11 $$TP{R}_{test}\left({\boldsymbol{Y}},\widehat{{\boldsymbol{Y}}}\right)={R}_{test}\left({\boldsymbol{Y}},\widehat{{\boldsymbol{Y}}}\right)$$

**Table 2:** Patch U-Net performance on binary segmentation.

Fold resolution of images during training	Dice coefficient on test set at full resolution	IoU	Precision	Recall	AUC
0.2	0.541	0.278	0.431	0.799	0.970
0.3	0.877	0.452	0.890	0.920	0.989
0.5	0.864	0.426	0.959	0.773	0.965
1	0.875	0.435	0.938	0.817	0.974

Model training was performed at different image resolutions with model performance reported as Dice coefficient^32,33^, IoU^36^, Precision^37^, Recall^37^, and AUC^38,39^ on the test set at full image resolution.

This was the *True Positive Rate (TPR)* and was used in the final metric *AUC* in Eq. (13).

12 $$FP{R}_{test}(Y,\widehat{Y})=\frac{1}{n}{\sum }_{i\in \{1,...,n\}}\frac{{\sum }_{h,w}{Y}_{i,h,w}^{\perp }\;.{\widehat{Y{\prime} }}_{i,h.w}}{{\sum }_{h,w}{Y}_{i,h,w}^{\perp }\;.{\widehat{Y{\prime} }}_{i,h.w}+{\sum }_{h,w}{Y}_{i,h,w}^{\perp }\;.{\widehat{Y}{\prime} }_{i,h,w}^{\perp }}$$

where $h,w,{{\boldsymbol{Y}}}^{\perp },i,\widehat{{\boldsymbol{Y}}{\prime} },{\widehat{{\boldsymbol{Y}}}{\prime} }^{\perp }$ have the same definitions as in Eqs. (8–10). This was the *False Positive Rate (FPR)* and was used in the final metric *AUC* in Eq. (13).

Employing an alternative threshold, such as 0.75 as opposed to the conventional 0.5, to discretize the variable $\widehat{Y}$ to get $\widehat{{\boldsymbol{Y}}{\prime} }$ and consequently ${\widehat{{\boldsymbol{Y}}}{\prime} }^{\perp }$ results in disparate *TPR* and *FPR* values across distinct threshold levels. The Receiver Operating Characteristic (ROC) curve^38,39^ is defined as the graphical representation formed by plotting TPR on the y-axis against FPR on the x-axis across various thresholds. In this instance, these thresholds are specifically delineated by all unique values within the interval [0,1] observed in $\widehat{Y}$ prior to the binarization process. This depiction offers a comprehensive visualisation of the nuanced trade-offs between these two performance metrics.

13 $$AUC=Area\;under\;ROC\left(TPR\;vs.FPR\right)$$

*AUC* was our final metric for evaluating model performance and was reported as *AUC*^38,39^ in Table 2.

### Model implementation

#### Optimiser

To optimise the model’s parameters, we employed the Adam optimiser^40^ with an initial learning rate of 0.001. Then, we decreased the learning rate according to an exponential schedule with a decay rate of 0.95 every 50 epochs.

#### Regularisation

To prevent overfitting, we employed the following regularisation technique: L2 weight decay^41^. L2 weight decay with a coefficient of 0.0001 was used to penalise large weights and encourage the model to arrive at sparse solutions.

#### Training procedure

Our training process consisted of 750 epochs for each experiment giving the model sufficient time to converge, each epoch containing 1000 random samples. We used a batch size of 50, and the model’s parameters were updated with mini-batch gradient descent.

#### Hardware and software setup

The model was built in Python 3.10.8. TensorFlow, a library developed to solve deep learning problems, was incorporated to increase model scalability, speed, and accuracy. Keras was used as a Python interface to TensorFlow. The following libraries and their required versions used in our network were as follows: keras 2.6.0, keras-preprocessing 1.1.2, numpy 1.19.5, tensorboard 2.6.0, tensorflow 2.6.0, scikit-image 0.18.1, tqdm, scipy, seaborn, and scikit-learn. Experiments were conducted on the following machines: MacBook Pro Apple with M1 Max Chip with 10-core CPU and 32-core GPU, HPC Hemera at HZDR on a Nvidia Tesla A100 GPU 40GB, HPC at ZIH TU Dresden on a NVIDIA A100-SXM4 Tensor Core-GPU.

## Data Records

### Data storage

The dataset is publicly available at the Rodare data repository^42^. Images were captured at the clinic and anonymised using an allocated study number. Images were stored on-site in secure UCL storage. All patient data and manual microscopy reports were entered on an encrypted database on a secure server in compliance with General Data Protection Regulation. This clinical database is NHS approved and procured, and regularly backed up.

### Data structure

The dataset is organised into three root folders: image, binary mask, and multi-class mask (Table 1). Each folder has 300 files in TIFF format and labelled incrementally.

### Demographics and symptomatology

**Fig. 2:**
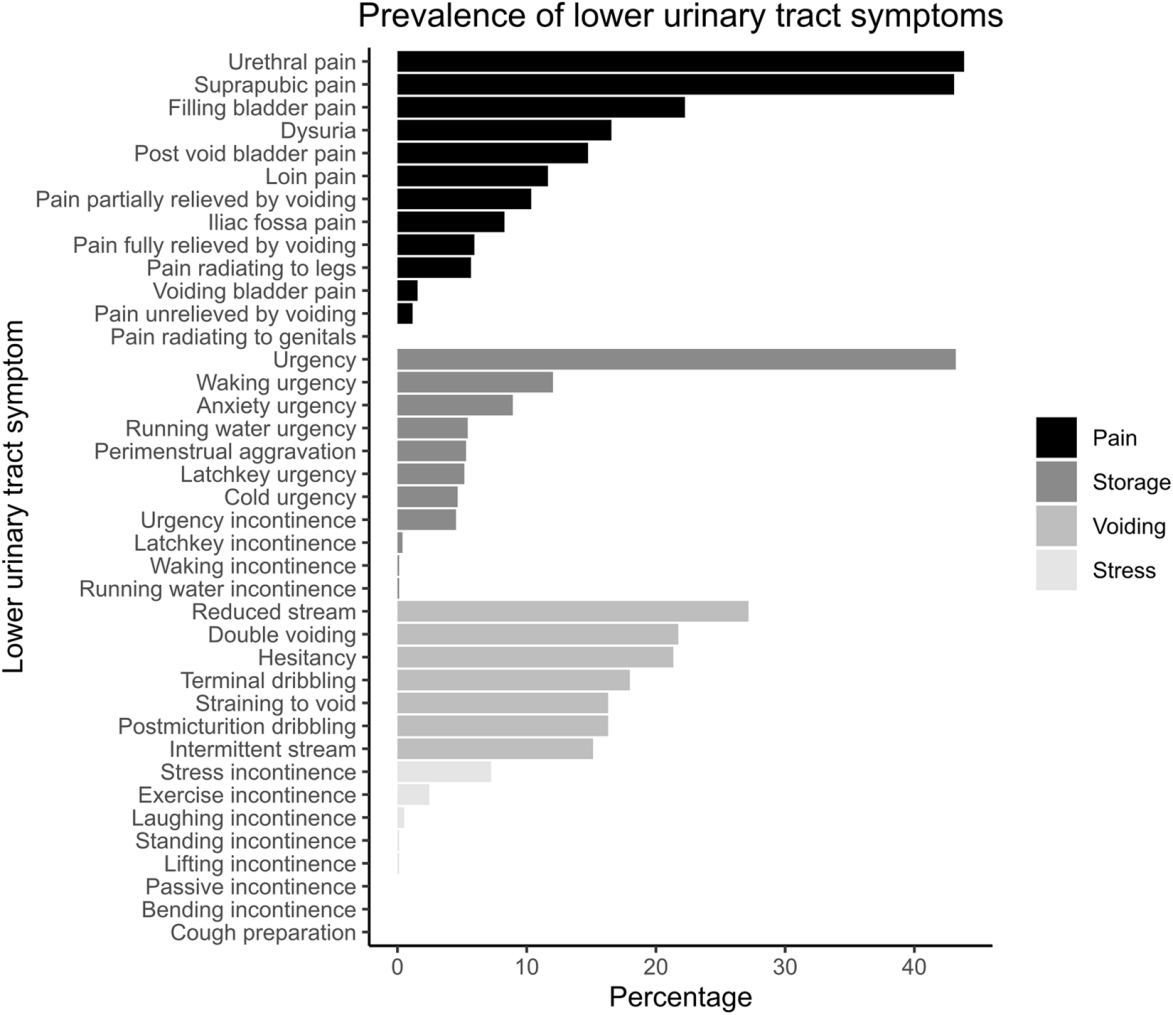
Prevalence of lower urinary tract symptoms (LUTS). Pain was the most common symptom associated with this cohort of patients, followed by storage and voiding. This is in keeping with the symptoms most predictive of microscopic pyuria and, in turn, UTI^15^.

The image dataset was obtained from urine samples of patients with symptomatic UTI. 300 patients (mean 42 ± 15 years, 95.1% female) were recruited. Patients reported a total of 3 LUTS (IQR 1–6 symptoms), with pain being the predominant symptom (median pain score 2, IQR 0–3), followed by storage, voiding, and stress (Fig. 2). Most samples contained WBC (median 6, IQR 2–22 WBC per 1 µl urine) and EPC (median 14, IQR 4–42 EPC per 1 µl urine), and were negative for RBC.

## Technical Validation

### Binary semantic segmentation using a neural network with random patch generator

**Fig. 3:**
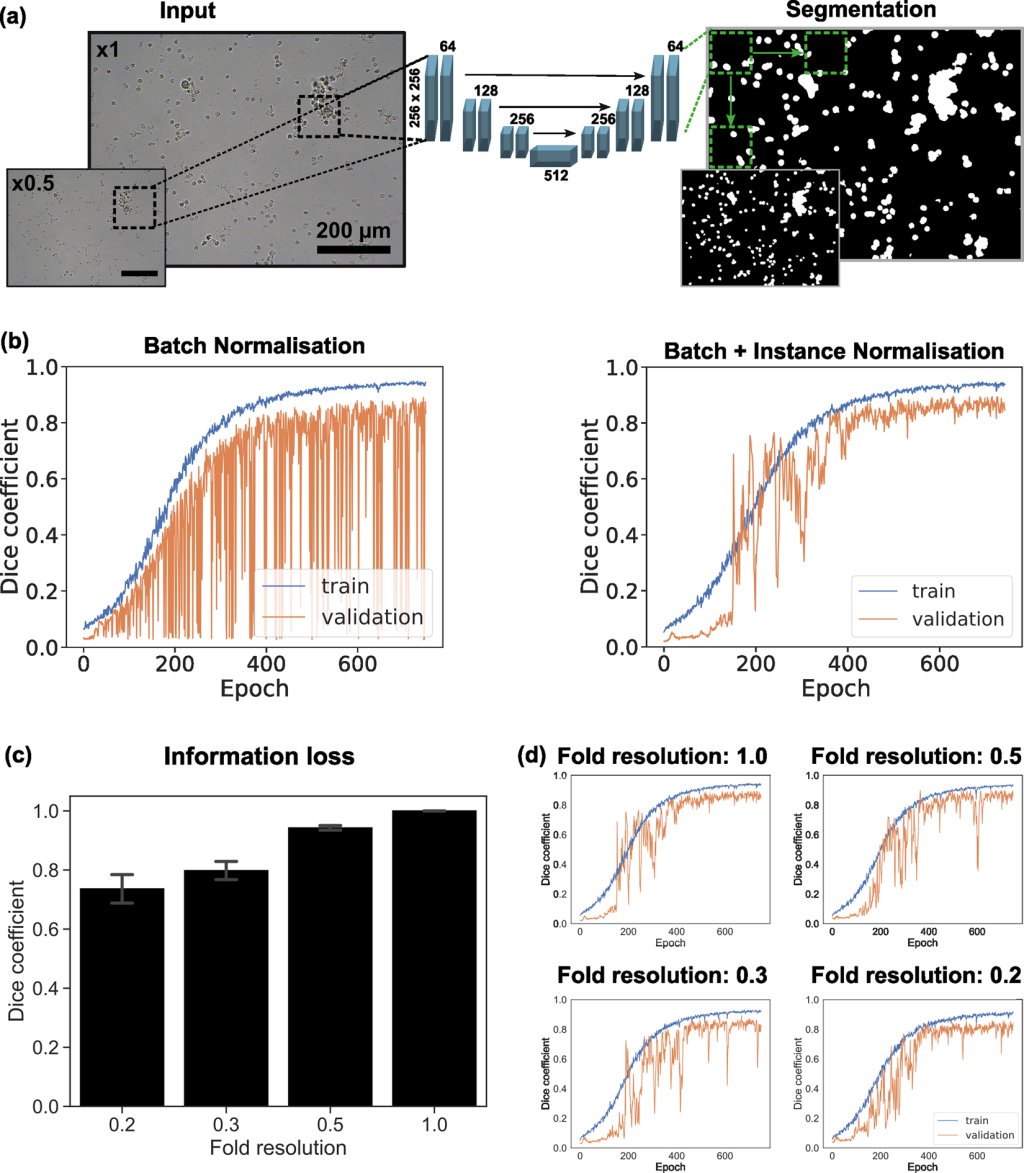
Binary Segmentation using Patch U-Net. (**a**) Patch U-Net architecture with patches as input and output resolutions 1.0 and 0.5, (**b**) Training performance with batch normalisation only (left) and combined batch and instance normalisation (right), (**c**) Information loss measured as Dice coefficient (±standard deviation) between original and downscaled-upscaled images, (**d**) Training and validation performance using original images at full resolution 1.0 and reduced resolutions at scale factors of 0.5, 0.3 and 0.2.

To evaluate the applicability of the dataset to deep-learning-based image segmentation, we developed a patch-based U-Net (Patch U-Net) similar to several other architectures proposed previously^27,43^ to perform urinary cell identification by binary semantic segmentation. The architecture of the proposed model incorporates a unique random patch generator (Fig. 3a) to produce multiple input and output patches at different resolutions in the requisite square-shaped U-net dimensions for data augmentation. The image and binary mask components of the dataset were equally and randomly split into train, validate, and test subsets with 100 images each. We chose this data split, as opposed to the conventional 70/20/10 split, to mitigate potential underrepresentation of certain cell types that are morphologically distinct, sparse, and yet significant.

Patch U-Net, which processes patches rather than whole images, was employed since preserving resolution was critical for detecting small objects such as bacteria. However, the dimensions of our input images were (1392, 1040, 1) making it computationally very expensive to process entire images. Employing a Patch U-Net was also effective since our dataset is sparse in nature. Thus, the model can converge faster when shown data that is relevant for semantic segmentation rather than the background. For this a filter was applied to the generated patches, where a batch of patches of shape (batch size, 256, 256, 1) was used for training only when a specific criteria (see section Batch generation - Methods) was satisfied.

### Impact of data normalisation on binary segmentation

During the initial stages of our experiments, an issue appeared involving the instability of validation accuracy during training. At times, the model displayed an unusual behaviour, classifying entire images as either foreground or background, resulting in a significant drop in accuracy. Although this behaviour tended to persist for only a few epochs, it raised concerns. To tackle this problem, instance normalisation layers were incorporated into our network architecture after each convolutional and deconvolutional block. These layers played a crucial role in preventing instance-specific mean and covariance shifts, thereby simplifying the learning process. This technique, introduced by Ulyanov *et al*.^28^, effectively alleviated the instability observed during training (Fig. 3b).

### Impact of image resolution on binary segmentation

To increase context within the same patch size while maintaining the same computational complexity, we tested the effects of reduced image resolutions. Such an approach is widely used in computer vision to increase computational efficiency. Specifically, we considered the resolutions at scale factors of 0.2, 0.3, 0.5 and 1 of the original resolution, referred to as fold resolution in Fig. 3. Notably, pixel information was lost during downscaling and consequent upscaling from any scale factor other than 1 (Fig. 3c). This loss of information was measured as the average Dice coefficient between original images at full resolution i.e. scale factor 1 and the corresponding images scaled down to a lower resolution. For example, images reduced to a factor of 0.2, 0.3 or 0.5 in scale were then scaled back up to full resolution. Pixel information was increasingly lost as the scale factor decreased as seen in Fig. 3c. The impact of resolution should therefore be carefully considered for this dataset.

To investigate if training on low resolution image and inference on high resolution image could serve as a viable alternative, we trained binary segmentation models on scale factors of 0.2, 0.3, 0.5 and 1. Figure 3d shows the training performance of our model using different images at scale factors of the original resolution to generate patches. Validation was performed on similar patches of the respective downscaling factor from the validation set. Once trained, inference was performed on the full resolution images from the test set (Table 2). Remarkably, results of all evaluating metrics suggested that a model trained on images downsampled as high as factor 0.3 of the full resolution may be as effective in inference on full resolution images, as the model trained on full resolution images.

### Multi-class morphological feature projection

To make our dataset applicable for computer vision tasks such as multi-class segmentation, object-detection and clustering, we have annotated the binary masks into seven classes (see section Dataset annotation - Methods). Multi-class segmentation annotations can be translated into object-detection annotations. This can be achieved by treating binary masks as a set of connected components on a black background and obtaining bounding boxes of each connected component.

**Fig. 4:**
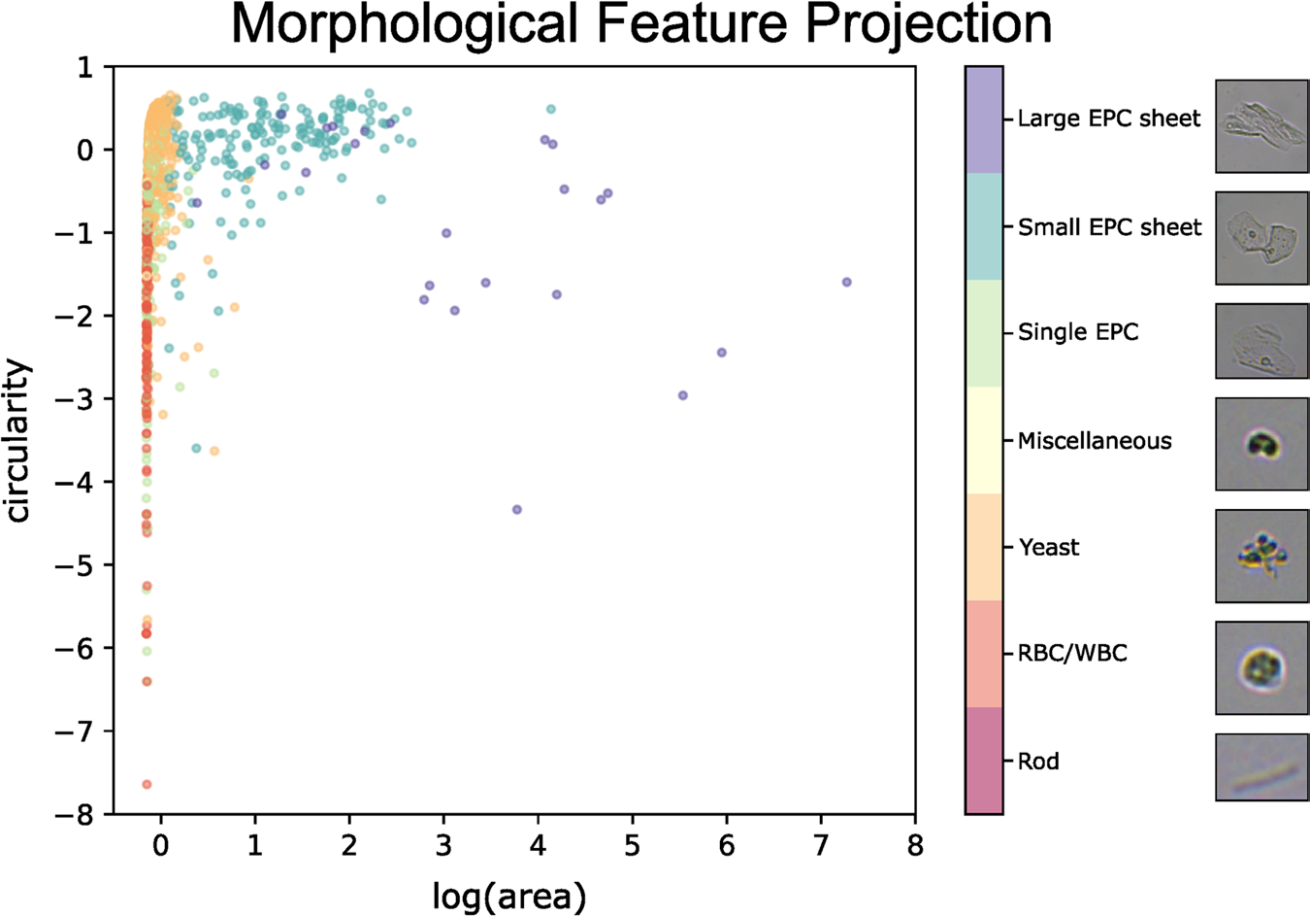
Morphological Feature Projection. 2D scatter plot of logarithm area vs. circularity measurements of connected components of interest extracted from binary segmentation (see Fig. 3). Data points are coloured by class according to their corresponding cell type.

To examine properties of the multi-class objects in an interpretable manner, we evaluated projections of some morphological features which we found to be particularly distinct. Specifically we evaluated area (µm) and circularity (value between 0.0 to 1.0, where 1.0 represents a perfect circle). We also scaled these values further using a standard scaler^44,45^. These are informative particle metrics in microscopic object analysis associated directly with the nature of the object. For this we first obtained connected components from the pixel-level multi-class masks present in the dataset. Next, the connected components were projected as manually labelled classes using a scatterplot with both features scaled, and area additionally log transformed (Fig. 4). Examples of each cell category are demonstrated in the legend.

In summary, UTI is a rising global problem and current diagnostic tests perform poorly. Here, we present an annotated, clinically-relevant, image dataset to perform binary and multiclass segmentation and object detection. We demonstrate the applicability and real world potential of deep learning to this clinical problem by training a simple semantic segmentation model. Moreover, we explore and present the effect of data normalisation and image resolution on model performance. This proof-of-concept dataset represents the initial steps towards a more fit for purpose and equitable diagnostic test for UTI.

### Supplementary information

Supplementary File 2

Supplementary File 1

## Data Availability

All code is available from https://github.com/casus/UMOD under MIT open source licence.
