# Supplementary material for: A clinical microscopy dataset to develop a deep learning diagnostic test for urinary tract infection
Source: Sci Data. 2024 Feb 1;11:155. doi: 10.1038/s41597-024-02975-0 (PMC10834944; doi:10.1038/s41597-024-02975-0)
Supplement: Supplementary file 1 — Supplementary File 2 [file 41597_2024_2975_MOESM1_ESM.docx]

**Appendix: List of abbreviations**

| **Abbreviation** | **Definition** |
| --- | --- |
| AUC | Area Under the ROC Curve |
| BIIG | Bladder Infection & Immunity Group |
| CPU | Central Processing Unit |
| Dice coefficient | Sørensen–Dice coefficient |
| EPC | Epithelial cells |
| FPR | False Positive Rate |
| GPU | Graphic Processing Unit |
| HCRW | Health and Care Research Wales |
| HPC | High Performance Computing |
| HRA | Health Research Authority |
| HZDR | Helmholtz-Zentrum Dresden-Rossendorf |
| IoU | Intersection over Union |
| IQR | Interquartile range |
| IRAS | Integrated Research Application System |
| keras | Python library |
| L2 | L2-norm |
| LED | Light emitting diode |
| LUTS | Lower urinary tract symtoms |
| NHS | National Health Service |
| numpy | Python library |
| POCT | Point-of-care testing |
| RBC | Red blood cells |
| REC | Research Ethics Committees |
| ReLU | Rectified Linear Unit |
| ROC | Receiver Operating Characteristic |
| scikit-image | Python library |
| scikit-learn | Python library |
| scipy | Python library |
| seaborn | Python library |
| TIFF | Tag Image File Format |
| TPR | True Positive Rate |
| tqdm | Python library |
| UTI | Urinary tract infection |
| WBC | White blood cells, or pyuria |
| ZIH TU Dresden | Zentrum für Informationsdienste und Hochleistungsrechnen, Technische Universität Dresden |
