# Supplementary material for: A clinical microscopy dataset to develop a deep learning diagnostic test for urinary tract infection
Source: Sci Data. 2024 Feb 1;11:155. doi: 10.1038/s41597-024-02975-0 (PMC10834944; doi:10.1038/s41597-024-02975-0)
Supplement: Supplementary file 2 — Supplementary File 1 [file 41597_2024_2975_MOESM2_ESM.pdf]

Study Number: Date:

Lower urinary tract symptoms (LUTS)<br>There are **39 questions**. Please circle your answer (Yes / No)		
**Storage symptoms**		**Circle**
1. Urgency	Do you have to hurry to pass urine because you might wet yourself?	Y / N
2. Urge incontinence	Do you hurry to pass urine but not make it in time?	Y / N
3. Latchkey urgency	Do you find that you have to rush to pass urine when you put a key in the front door?	Y / N
4. Latchkey urgency incontinence	If you have to rush to pass urine when you put a key in the front door, do you ever leak with this?	Y / N
5. Waking urgency	Do you have to hurry to pass urine because you might wet yourself when you wake up in the morning?	Y / N
6. Waking urge incontinence	If you have to hurry to pass urine when you wake up in the morning, do you ever wet yourself?	Y / N
7. Running water urgency	Do you have to hurry to pass urine because you might wet yourself on hearing running water?	Y / N
8. Running water urge incontinence	If you have to hurry to pass urine on hearing running water, do you ever wet yourself?	Y / N
9. Cold urgency	Do you have to hurry to pass urine because you might wet yourself on exposure to cold?	Y / N
10. Anxiety urgency	Do you have to hurry to pass urine because you might wet yourself on when you are worried or anxious?	Y / N
11. Premenstrual aggravation	Do you have to hurry to pass urine because you might wet yourself, more around the time of your period?	Y / N
**Stress symptoms**		**Circle**
12. Cough sneeze incontinence	Do you leak on coughing or sneezing?	Y / N
13. Exercise incontinence	Do you leak on exercise?	Y / N
14. Laughing incontinence	Do you leak on laughing?	Y / N
15. Passive incontinence	Do you leak for no good reason?	Y / N
16. Bending incontinence	Do you leak on bending?	Y / N
17. Standing incontinence	Do you leak on standing from sitting?	Y / N
18. Lifting incontinence	Do you leak on lifting anything?	Y / N
19. Pre-cough preparation	Do you get ready to avoid leaking when you are about to cough?	Y / N

Study Number: Date:

Voiding symptoms		Circle
20. Hesitancy	Is to slow to start passing urine?	Y / N
21. Reduced stream	Is the urinary stream reduced?	Y / N
22. Intermittent stream	Does the stream stop and start?	Y / N
23. Straining to void	Do you have to strain to pass urine?	Y / N
24. Terminal dribbling	Does the stream dribble at the end?	Y / N
25. Postvoid dribbling	Does it dribble after you have finished?	Y / N
26. Double voiding	Do you pass urine, leave the toilet, and then have to go back again?	Y / N
**Pain symptoms**		**Circle**
27. Suprapubic pain	Do you get bladder pain felt over the pubic area?	Y / N
28. Filling bladder pain	Does the b ladder gain worse as the bladder fills?	Y / N
29. Voiding bladder pain	Do you get bladder pain during voiding?	Y / N
30. Post void bladder pain	Do you get bladder pain after voiding?	Y / N
31. Pain relieved by voiding	Do you get bladder pain fully relieved by voiding?	Y / N
32. Partially voided relief	Do you get bladder pain partially relieved by voiding?	Y / N
33. No voiding relief	Do you get bladder pain partially unrelieved by voiding?	Y / N
34. Loin pain	Do you get flank pain in the kidney area?	Y / N
35. Iliac fossa pain	Do you get pain to the right or left of the low abdomen?	Y / N
36. Pain radiation to genitals	Do you get pain radiating into the vagina?	Y / N
37. Pain radiation to legs	Do you get pain radiating down your legs?	Y / N
38. Dysuria	Does it burn when you pass urine?	Y / N
39. Urethral pain	Do you experience pain the urethra (the tube through which the urine passes?	Y / N
